# Sites and Population in London

**Published:** 1903-03-07

**Authors:** 


### HOSPITAL ADMINISTRATION.

### CONSTRUCTION AND ECONOMICS.

### SITES AND POPULATION IN LONDON.

### UNEQUAL DISTRIBUTION OF HOSPITALS IN THE METROPOLIS.

Suggested King's-Westminster and Herring-Miller Amalgamations.

An influential and representative gathering and a full attendance were noticeable at the meeting held at the Caxton Hall, Westminster, on Tuesday last, under the auspices of the Hospitals Association, when a paper was read by Sir Henry Burdett on "Hospital Sites and Population in London," and a discussion took place on the question "Ought any, and if so which, of the large hospitals to be moved from their present sites to new ones situate on (*a*) more congested districts, or (*b*) the country?"

Sir Trevor Lawrence, Bart., K.C.V.O., the treasurer of St. Bartholomew's Hospital, presided.

Sir Henry Burdett's Paper.

Sir Henry Burdett commenced his address by explaining the method he had adopted of dividing up London into postal districts upon a map, showing the situation of each hospital, with a view to arriving at the proportion of hospital beds to population in each of these districts.

Sir Henry Burdett showed that in District (1), the City of London, the ratio of beds to population in 1901 was as 1 to 40; District (2), Stepney, Shoreditch, Finsbury, Holborn, City of Westminster, Southwark and Bermondsey, it was as 1 to 418; District (3), Poplar, Bethnal Green, Hackney, Islington, St. Pancras, St. Marylebone, Kensington, Paddington, Chelsea, Battersea, Lambeth, Camberwell, and Deptford it was as 1 to 1,054; District (4), Stoke Newington, Hampstead, Fulham, Wandsworth, Lewisham, Greenwich, and Woolwich, it was as 1 to 1,877; District (5), West Ham and Romford, Tottenham and Edmonton, Barnet, Hendon, and Brentford, Croydon, Kingston, and Richmond, Bromley and Dartford, it was as 1 to 2,482.

Dealing with the districts as a whole, the speaker showed that a population of 4,715,016 in 1881 has increased to 6,276,812 in 1901—that is, at the rate of a little over 33 per cent. in 20 years. The ratio of beds to population is as 1 to 922.

In other words, seeing that the usual estimate in regard to hospital beds in general hospitals in relation to the needs of the population is usually taken at 1 to 1,000 people, we arrive at this splendid result, that Greater London is at present provided with an adequate number of such beds, without counting a single one of the 2,477 beds provided in addition by the special hospitals. Sir Henry said: "I have thought it well to bring this momentous fact out at the outset, because it disposes effectually of the pessimistic views which sometimes find expression in the Press in regard to the great voluntary hospitals, and proves to demonstration that, apart altogether from considerations of high policy which must negative the idea of municipal hospitals where the people of a great nation are well educated and intelligent in regard to their own best interests, the needs of the population do not require any such additional hospital aid at the present time, so far as the Metropolis of the Empire is concerned."

Sir Henry explained his reasons for dividing London in the manner he had done to demonstrate his contentions and discussed many importants factors of the situation, after which he proceeded to particularise the three districts without sufficient hospital accommodation, namely, South London, Tottenham, and Battersea. Tottenham, he explained, although it had a hospital for the use of its present population, was to receive an addition of 40,000 residents, sent there by the London County Council. He urged that the County Council were under the obligation to see that these people could secure proper care when ill. He proposed that they should be asked to make a loan to the Tottenham Hospital authorities, to be repaid with interest by a sinking fund. This loan was to secure further wards on a site available at the Tottenham Hospital, and to be at the service of the new residents and look to them for maintenance. In the matter of South London he referred to the report that a scheme was under consideration to supply the needs of South London by removing King's College Hospital to Camberwell. Deptford, Woolwich, and Greenwich would still remain inadequately provided for, and to meet this want he suggested that the North London Hospital might amalgamate with the Miller Hospital at Greenwich, and the institution might be named the Herring-Miller Hospital. He pointed out that the North London Hospital is in need of modern buildings if it remains where it is, but at the same time the district was sufficiently provided for without it. Lastly, he dealt with the needs of Battersea, and said that the Bolingbroke Hospital was prepared to extend and build a hospital suitable to the requirements of the district, if support were forthcoming. Having thus drawn up a proposal for the provision of adequate hospital provision throughout the metropolis, Sir Henry turned to the question whether any of the larger hospitals might suitably move from their present sites to more congested districts. He thought that such proposals could only be considered in regard to King's College Hospital, the Westminster, and St. George's Hospitals. King's College he had already referred to. Although it would undoubtedly be a good thing for Battersea if the Westminster Hospital could move there and amalgamate with the Bolingbroke Hospital, still the financial difficulties were very great, and it seemed unlikely that enough money could be obtained from the sale of the present site to pay for the removal. As to St. George's, he could bring forward nothing in favour of removal. As to the question of St. Bartholomew's he said that it was not ripe for public discussion, as the results of the Committee of Inquiry were not fully announced.

The Discussion.

Mr. Sydney Holland opened the discussion by demurring at the method Sir Henry Burdett had adopted in dividing London, in as far as it related to East London, and the provision of hospital beds for it. He thought that chronic cases should be relegated to Poor-law infirmaries, and that the voluntary hospitals could not be expected to provide for them. He entirely differed from Sir Henry's contention that Poor-law infirmaries would provide useful material for medical education. What on earth could a student learn at a Poor-law infirmary? Sir Henry's proposal in regard to the London County Council and the Tottenham Hospital was practically advocating rate support for the hospitals. Mr. Holland strongly advocated the amalgamation of King's College and the Westminster Hospitals on a site in the suburbs—they could do their good work equally well in the suburbs—and, he would suggest, under the name of The King's Westminster Hospital. They all knew that the hospital staffs objected to moving, and that constituted the chief difficulty.

Sir Henry Burdett explained that the Eastern district on his map covered Poplar, Stepney, and Bethnal Green, purely arbitrary postal-district divisions, covering lesser London with about 4½ millions. People frequently went to hospitals from long distances, and it was impossible to define in Mr. Holland's sense the districts provided for by a particular hospital in London. A man at Islington might go to St. George's Hospital, for instance, passing hospitals on the way—where he could have had the services of the same practitioners —from some powerful or sentimental reason, so that every hospital served more or less than the population around it. As to the suggestion that what he proposed would make Tottenham a municipal institution, he entirely disagreed. This was an altogether exceptional case. The County Council were taking 40,000 people out there, and not making any provision whatever for their hospital accommodation, and all they were asked to do was to make a loan which could be repaid out of a sinking fund with interest. As to the Poor-law infirmaries, the medical student would learn much there that he needed but could not learn in the general hospitals, which would be of immense service to him in general practice, where the majority of cases would be of a simple nature.

Mr. Beattie Campbell raised the objection that many subscribers to hospitals would not continue to support them if moved to other localities or amalgamated with other institutions. He thought, on sanitary grounds, it would be a good thing to remove King's College Hospital, but the difficulties of securing support under new conditions should not be lost sight of.

Mr. F. S. Franklin questioned Sir Henry Burdett on the attitude likely to be adopted by King Edward's Fund in regard to proposed removals, but this the Chairman ruled out of order.

Mr. Conrad Thies warmly thanked Sir Henry Burdett for his able and interesting paper. He said that whenever the subject of the provision of hospitals in relation to population was under discussion, the accommodation provided by the Poor-law and Metropolitan Asylums Board should be taken into calculation. Many of their institutions were now so excellent as to equal the best under the voluntary system, and he, with Sir Henry Burdett, regretted that they could not be utilised for medical education. He deprecated any attempt to check the influx of country patients to the London hospitals, as he thought the country had as much right to the best treatment as the town dwellers, and that this material being always of a special nature was of use to the medical student. He advocated the establishment of a large central hospital in the country where serious town cases might be treated, with a view to proving whether treatment in pure air was not greatly beneficial. In respect to the Tottenham Hospital question, he thought such a scheme would entail representation on the administration of voluntary hospitals by the County Council, and so give them control. He expressed appreciation of the authority—made possible by the power of the purse—which the King's, the Sunday and Saturday Funds were exercising, to inforce their recommendations He would like to see this authority so effective as to be able to check the establishment of unnecessary and undesirable institutions.

Dr. Gilbart Smith said there had never been a more important subject discussed by that Association, nor one that had had so fair a hearing. But he thought they had omitted to consider one aspect of the case for moving, and that it was not so much the needs of the people in the outlying districts that would be served as the needs of the hospitals themselves for expansion. Modern requirements were so great that he was certain that during the next twenty years the great hospitals themselves would come to the conclusion that their buildings were not suitable, and no longer permitted of proper development. He thought St. Bartholomew's was a case in point, and it was with a view to future necessities that he favoured a removal, say, to St. Luke's. As to Tottenham he thought it as much the duty of the L.C.C. to provide beds for the people they were moving as to provide amusements and bands on the Embankment. Another point brought out by the study of the situation of the Tottenham Hospital was the fact that it was officered from London, showing that it was not impossible to get medical men to serve outlying hospitals. One of the difficulties of the present crowded state of the central hospitals was the tendency to expel certain diseases—typhoid was a case in point—the study of which was necessary to fulfil the medical curriculum. There were also many forms of disease which could now only be studied at the Poor Law infirmaries, and he would welcome any scheme which would throw these and the fever hospitals open for purposes of medical education. In concluding Dr. Gilbart Smith expressed a hope that the present discussion would lead to a real issue on the important questions involved.

Sir Edmund Hay Currie said the question of moving voluntary hospitals must be dealt with in a very gentle manner, and that great consideration should be given to the views of those who supported them; if something could be done to meet this it would be one of the greatest blessings that could fall upon the country. They were all greatly indebted to Sir Henry Burdett, than whom no one had done more for the hospitals, for his most able paper.

Sir Trevor Lawrence said he did not altogether agree with the views that had been expressed regarding the distribution of population. That was a subject that had been discussed at considerable length in connection with the suggested moving of St. Bartholomew's. They must remember that the census was taken on Sunday night when everyone who could get out of London was away. If it had been taken on Monday morning the position of affairs would have been found to be totally different. One consideration with regard to the medical staffs they must not lose sight of —the desirability of economising the time of the distinguished physicians and surgeons who gave their services. It would be scarcely possible for these eminent men to continue to serve them if they moved into the suburbs. They heard a great deal about the requirements of the experts, but both University College and Charing Cross Hospitals were being rebuilt on small areas, and if the present views of experts were to prevail this would never have been done. They were told at St. Bartholonew's that it was a crime to build on such valuable land; but what better use could be made of a site than the saving of human life? In regard to the question of removing St. George's, he said he could not conceive of a better site for a hospital. King's College Hospital was cramped for room, and so, no doubt, was Westminster; but they must not forget the enormous cost of moving and the serious sacrifice of money involved. In conclusion he referred to St. Bartholomew's Hospital, and said he was thankful to say that the committee appointed to consider the question of the removal of St. Bartholomew's had, by a majority of 14 to 1, decided to retain the present site.

Sir Timothy Holmes moved a vote of thanks to Sir Henry Burdett for his admirable and thoughtful paper. As to St. George's he could only say that they had been so strongly urged to move that they had thought it necessary to bring the matter before the public. He could say no more on the subject till that meeting had been held.

Mr. Michelli seconded the vote in eulogistic terms.

In reply, Sir Henry Burdett dealt with the points that had been raised during the discussion. Referring to Mr. Beattie Campbell's remarks he said that, while prepared to treat with due consideration the feelings of those who found the money for the hospitals, they must not be alarmed at meeting with personal idiosyncracies in individual subscribers. Since Mr. Beattie Campbell was present and had spoken on the removal of other institutions, he felt he might point out that the Royal Westminster Ophthalmic Hospital, with which Mr. Campbell was connected, was one of those hospitals which might with advantage be amalgamated with one of the other kindred institutions and moved from its present site, so that the Charing Cross Hospital might acquire its land, which he believed it was willing to buy. (Hear, hear.) As to the fresh-air argument it would be utterly wrong as a general principle to move out on that ground alone. Many of the general hospitals had country branches to provide for convalescent cases. Dealing with Sir Edmund Currie's objection to bring pressure on a particular institutions to move, Sir Henry said he knew Mr. George Herring, who had done yeoman service to the London hospitals, and in making the suggestion as to the "Herring-Miller" amalgamation, he did it as a compliment to him. He knew there was a question of rebuilding the North-West London Hospital, that it could be moved from its present site, and that there were enough beds in the district without it. If the amalgamation with the Greenwich hospital could be arranged it would do great good to both institutions and to London, and he hoped the scheme would have Mr. Herring's sympathy. Referring again to Tottenham, he said the principle of accepting money from public bodies had already been accepted since contributions were received from Boards of Guardians. In the case of Tottenham a great emergency had to be met, and would probably never occur again. All that was proposed was that the L.C.C. should lend a sum of money to construct the buildings necessary to provide hospital accommodation for the 40,000 people they were bringing out, which could be repaid with interest by means of a sinking fund. Such a special arrangement in the peculiar circumstances would not bring them one step nearer to municipal control.

He concluded by moving a vote of thanks to the chairman, which was seconded by Mr. Bryant and carried by acclamation.

